# Effect of intervention on preventive practices of hypertension and diabetes among teachers

**DOI:** 10.4102/jphia.v17i1.1418

**Published:** 2026-02-03

**Authors:** Victoria O. Rotimi-Oyedepo, Patience E. Samson-Akpan, Mary A. Olofin-Samuel

**Affiliations:** 1 Department of Community Nursing, School of Nursing, Babcock University, Ogun State, Nigeria; 2 Department of Nursing, Faculty of Nursing, University of Calabar, Calabar, Nigeria; 3 Department of Nursing, School of Nursing, Babcock University, Ilishan Remo, Nigeria; 4 Department of Nursing, Faculty of Basic Medical Sciences, Ekiti State University, Ado-Ekiti, Ekiti State, Nigeria

**Keywords:** educational intervention, preventive practices, hypertension, diabetes, teachers

## Abstract

**Background:**

This study addressed the critical gap in teachers’ preventive practices regarding hypertension and diabetes, highlighting the need for workplace health interventions to improve disease prevention.

**Aim:**

This study aimed to examine the effect of an educational intervention on self-reported preventive practices of hypertension and diabetes among teachers in selected schools in Lagos State.

**Setting:**

The research setting comprised Lagos Island and Yaba Local Government Areas (LGAs) in Lagos State, Nigeria, both of which are historically significant and serve as key centres for education and commerce.

**Methods:**

A quasi-experimental research design was adopted, involving an experimental group that received the intervention and a control group that did not. The study population comprised public secondary school teachers, with a total of 176 participants, selected using a multistage sampling procedure. Data were collected using a structured questionnaire adapted from the Health-Promoting Lifestyle Profile II. Descriptive and inferential statistical analyses were conducted.

**Results:**

There was no significant difference in self-reported preventive practices between the experimental and control groups before the intervention (Cohen’s *d* = –0.0072, *p* = 0.953). However, post-intervention results showed a significant improvement in the experimental group compared to the control group (Cohen’s *d* = 12.41, *p* < 0.001).

**Conclusion:**

The educational intervention implemented in this study significantly enhanced teachers’ preventive practices regarding hypertension and diabetes. It recommends that schools should collaborate with health agencies to conduct routine health screenings and awareness programmes.

**Contribution:**

This study provided empirical evidence that the educational intervention significantly improved teachers’ self-reported preventive practices for hypertension and diabetes, reinforcing the need for workplace-based health promotion.

## Introduction

Non-communicable diseases (NCDs) represent a serious global health concern, contributing substantially to morbidity and death rates globally. The World Health Organization (WHO) reported that NCDs accounted for 74% of worldwide fatalities in 2022, with cardiovascular diseases and diabetes mellitus at the forefront of these figures.^1^ Sub-Saharan Africa, particularly Nigeria, is witnessing a rapid increase in the incidence of NCDs, propelled by urbanisation, lifestyle modifications and insufficient understanding regarding preventative strategies. Non-communicable diseases in Nigeria constitute 29% of total mortality, with hypertension and diabetes being significant factors, responsible for 22% of fatalities among adults aged 30 to 69 years.^2^

The prevalence of NCDs in Lagos State, Nigeria’s most populous metropolitan centre, is notably concerning. The incidence of hypertension is 27.5%, while diabetes is at 4.8%, indicating an escalating public health concern.^2^ This escalating tendency is associated with urbanisation, heightened intake of processed foods, sedentary lifestyles and insufficient health education.^3,4^ Despite hypertension and diabetes being highly avoidable NCDs, insufficient information and the absence of proactive preventive measures perpetuate their elevated prevalence in Nigeria. Notwithstanding awareness initiatives, several individuals, especially teachers who significantly influence societal health behaviours and are key influencers in school-based health promotion, are uneducated on the risk factors, symptoms and preventative strategies for these illnesses.^5^

Educators, as pivotal figures in society, have the capacity to act as health advocates within their educational institutions and communities. Research reveals that educators frequently possess insufficient understanding about the prevention and treatment of NCDs, rendering them vulnerable to hypertension, diabetes and associated consequences.^6,7^ The disparity in teaching practices is affected by several variables, such as restricted access to specialised health education programmes, insufficient focus on NCD prevention in teacher training curricula and inadequate health promotion measures within schools.^8,9^ Inadequate self-efficacy in preventive activities may jeopardise instructors and hinder their ability to effectively impact pupils and the wider community about NCD prevention.

Yu et al.^10^ ascribed the elevated incidence of chronic illnesses in Africa to behavioural modifications, including sedentary lifestyles and diets rich in saturated fats, salt and sugar, which are shaped by industry, urbanisation and globalisation. Selvam et al.^8^ emphasised that obesity, smoking and excessive alcohol intake account for over 50% of the differences in hypertension and diabetes, with numerous individuals neglecting to check their blood pressure or adhere to a balanced diet. Kilama et al.^11^ discovered that 66.80% of residents in Kinondoni Municipality, Kimara, possessed broad awareness about hypertension and diabetes; however, only 19.75% recognised the related risk factors, with the use of fatty foods and stress identified as prevalent hazards. Michael et al.^12^ found that awareness of hypertension and diabetes was inadequate among hypertensive patients in Auchi, with just 18% identifying risk factors, and many exhibiting negative attitudes towards treatment and bad lifestyle practices. Mutyambizi et al.^13,14,15^ corroborated similar findings, indicating that just 41% of Nigerian adults possessed knowledge about hypertension and diabetes, with women exhibiting more awareness than men, and only 32.1% of patients adhering to their medication appropriately. These studies together emphasise the necessity for more public education on hypertension and diabetes, specifically about risk factors, lifestyle changes and adherence to treatment.

Despite persistent initiatives by the government and non-governmental organisations (NGOs) to combat NCDs, the majority of programmes emphasise clinical treatment over prevention.^16,17,18^ Moreover, current public health education campaigns mostly focus on healthcare professionals or the general population, overlooking the distinctive role that educators might have in facilitating behavioural change.^9^ It is important to incorporate NCD-focused educational interventions into teacher training and professional development programmes to promote proactive health behaviours.^19,20,21^

This study seeks to address this gap by assessing the effect of an educational intervention on teachers’ self-reported preventive practices regarding hypertension and diabetes in selected local government areas (LGAs) of Lagos State. The general objective of this study was to determine the effect of educational intervention on self-reporting preventive practices of hypertension and diabetes among teachers in selected schools in Lagos State. The specific objectives that guided the design and implementation of this study are to:

- Assess the pre-intervention level of self-reported preventive practices of hypertension and diabetes among teachers in selected LGAs of Lagos State.
- Assess post-intervention level of self-reported preventive practices of hypertension and diabetes among teachers in selected LGAs of Lagos State.

Two research hypotheses were formulated for this study:

- There is no significant difference between the mean scores of teachers in the experimental and control groups before intervention on self-reported preventive practices of NCD (hypertension and diabetes).
- There is no significant difference between the mean scores of teachers in the experimental and control groups after intervention on self-reported preventive practices of NCD (hypertension and diabetes).

## Research methods and design

### Study design

The study adopted a quasi-experimental research design, involving an experimental group and a control group, to assess the effect of an educational intervention on self-reported preventive practices of hypertension and diabetes among teachers in selected LGAs of Lagos State. The experimental group received an educational intervention, while the control group did not receive any intervention.

### Setting

Lagos State, Nigeria, serves as a dynamic research setting because of its geographical, economic and educational significance. Covering approximately 3577 km^2^, Lagos is a coastal state featuring major islands, lagoons and a tropical savanna climate that influences its environmental and urban dynamics. As one of Nigeria’s most densely populated and urbanised states, Lagos has a complex infrastructure, including extensive transportation networks, major seaports and an international airport. However, the rapid urban expansion has also led to challenges such as land scarcity, flooding and environmental degradation, prompting continuous urban planning and development efforts. Lagos’ strategic importance in trade and commerce, coupled with its diverse population of over 20 million people, makes it an ideal location for research focused on public institutions, including the education sector. Given its rich socio-economic diversity, the state provides a broad spectrum of study participants from varied cultural and professional backgrounds, making it a valuable site for educational research.

The research was conducted in public secondary schools across two LGAs: Lagos Island and Yaba. Lagos Island LGA, known for its historical and commercial importance, houses significant educational institutions such as Eko Boys’ High School and Methodist Boys’ High School, alongside 33 public and private secondary schools. Despite being a commercial hub, schools in Lagos Island face challenges such as overcrowding and infrastructural deficits, which impact teaching and learning. Similarly, Yaba LGA is a prominent educational centre, home to institutions such as the University of Lagos and Yaba College of Technology. The area boasts 29 secondary schools and a dedicated teaching workforce of 876 teachers, reflecting the local government’s commitment to quality education. The selection of these LGAs facilitated a structured research design, with schools in one LGA designated as the experimental group and those in the other as the control group, ensuring geographical separation to prevent study contamination.

### Study population and sampling strategy

**FIGURE 1:**
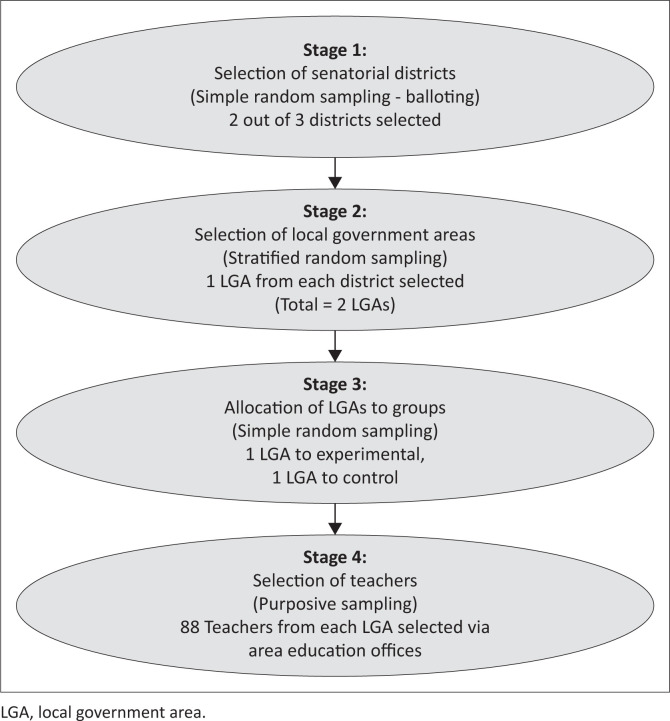
Flow chart of sampling technique.

The study population comprised all public secondary school teachers in Lagos State. Inclusion criteria included teachers with at least 3 years of experience, proficiency in English and willingness to participate, while those who were ill, pregnant, had undergone recent NCD prevention training or were on long-term leave were excluded. The sample size was determined using Lemeshow’s formula for paired *t*-tests, incorporating a 10% attrition rate, resulting in 176 participants. The sample was divided equally, with 88 participants in each group. A multistage sampling procedure was employed, beginning with selecting two out of three senatorial districts using a random sampling technique. One LGA from each selected district was then chosen using stratified sampling. These LGAs were assigned to experimental and control groups through simple random sampling. Finally, 88 teachers from each LGA were selected using purposive sampling, with the Area Education Office serving as the meeting point (see Figure 1).

### Intervention

Instructional Guide for the Educational Intervention was designed by the researchers to serve as guide. The researchers developed the instructional guide, which was also given to experts in the field of evaluation as well as experienced health practitioners, for vetting. It was trial tested to see the workability of the instruments. This aimed to improve the knowledge and practices of the participants towards hypertension and diabetes. The teaching exercise lasted 1 h during the intervention phase.

The training was conducted by trained research assistants, who were nursing officers, with the researcher actively participating in the teaching process and overseeing the proper execution of the intervention package and research protocols. The educational sessions were designed to address prevention practices related to hypertension and diabetes among teachers. The primary goal was to improve teachers’ understanding of NCDs and equip them with effective prevention strategies. The training was structured into modules, each lasting 1 h and conducted twice a week. The first module introduced participants to the research study, where research assistants formally welcomed them and provided an overview of hypertension, including its meaning, signs and symptoms, causes, risk factors and prevention strategies. Teaching aids such as pamphlets and printed materials were used to enhance comprehension. A question-and-answer session followed each training to clarify concepts, and participants engaged further through a WhatsApp platform created for continuous learning and reinforcement of the lessons.

The second module, delivered in the third week of the intervention, focused on diabetes, covering its definition, symptoms, causes, risk factors and preventive measures. Similar to the first module, instructional materials were provided, and interactive discussions were encouraged to ensure participants fully grasped the concepts. The WhatsApp platform continued to serve as a follow-up tool, reinforcing the key points covered in the training. At the end of the intervention, a post-test was administered to evaluate the impact of the training on participants’ prevention practices regarding NCDs. This assessment aimed to measure improvements and determine the effectiveness of the intervention in enhancing preventive behaviours among teachers.

Each session lasted 1 h, structured into two modules delivered twice weekly, covering hypertension in the first phase and diabetes in the second (second week). The curriculum content included definitions, symptoms, causes, risk factors and preventive measures of both conditions, reinforced through interactive discussions and a WhatsApp platform for continuous learning. Facilitators were qualified nursing officers with oversight from the researcher, while experts with master’s and PhD qualifications vetted the instructional guide to ensure content validity. The experimental group, made up of 86 respondents, was given pamphlets and printed materials as teaching aids.

### Data collection

The study employed a structured questionnaire adapted from the Health-Promoting Lifestyle Profile II (HPLP-II) by^22^ to collect data on preventive practices related to hypertension and diabetes. The instrument had two sections: Section A covered physical and physiological assessment of the respondents, Section B comprised 20 items assessing self-reported preventive practices and responses were measured on a four-point Likert scale.

Items on self-reported preventive practices of hypertension were a 10-item scale. An adapted Likert four scale, such as ‘Always – 4’, ‘Sometimes – 3’, ‘Rarely – 2’ and ‘Never – 1’, was used to measure the self-reported preventive practice of hypertension. Score between 35 and 40 was classified as good practice, 25–34 was classified as intermediate practice and less than 25 was classified as poor practice. Likewise, items on self-reported preventive practices of diabetes were a 10-item scale. Score ranging from 35 to 40 was classified as good practice, 25–34 was classified as intermediate practice and less than 25 was classified as poor practice.

Items on self-reported preventive practices of both hypertension and diabetes were a 20-item. Score between 70 and 80 was classified as good practice, 50–69 was classified as intermediate practice and less than 50 was classified as poor practice. The instrument’s validity was confirmed by experts in nursing science and measurement, ensuring content and face validity. The reliability was tested using the internal consistency method, yielding a Cronbach’s alpha value of 0.819 for preventive practices. The reliability coefficient indicates acceptable internal consistency. Data collection occurred in three phases: pre-intervention, intervention and post-intervention.

### Data analysis

The researcher numbered instruments for tracking, checked responses for accuracy and manually coded data before analysis. Statistical analysis was conducted using IBM Statistical Package for Social Sciences (SPSS) version 28, with descriptive statistics summarising variables through mean, median, standard deviation, frequency and proportions. Differences between groups were assessed using an independent *t*-test, and hypotheses were tested at a 0.05 significance level.

The following section presents the results and findings of the study.

### Ethical considerations

Ethical clearance to conduct this study was obtained from the Babcock University Health Research Ethics Committee (NHREC/25/12/2023) and Lagos State Ministry of Education Research. Participants were fully informed about the study’s purpose, procedures, potential risks and benefits, and their voluntary consent was obtained. Confidentiality and anonymity were ensured by replacing identifying information with numerical codes, while data security measures restricted access to authorised personnel only. Participation was entirely voluntary, with non-maleficence upheld to prevent harm to participants. The study prioritised beneficence, ensuring respect for participants and their best interests. Additionally, the research contributed to knowledge on the impact of educational interventions on teachers’ self-reported preventive practices regarding NCDs.

## Results

Out of the 176 participants projected for the study, only 169 participants (86 participants in the Experimental group, while 83 participants in the Control group) participated in all the stages of data collection, representing a 96.02% response rate.

The socio-demographic characteristics of the respondents in both the experimental and control groups reveal similarities and slight variations in key attributes (see Table 1). In terms of gender distribution, women constitute the majority in both groups, with 66.3% in the experimental group and 61.4% in the control group. Age-wise, a substantial proportion of respondents fall within the 35–44 years age range, representing 43.0% of the experimental group and 54.2% of the control group. Notably, there are more respondents aged 45 years and above in the experimental group (45.3%) compared to the control group (30.1%), indicating a slightly older demographic among the experimental group participants. Regarding religious affiliation, Christianity dominates both groups, with 59.3% in the experimental group and 65.1% in the control group, followed by Islam at 33.7% and 31.3%, respectively. Traditional and other religious affiliations have minimal representation in both groups.

**TABLE 1:** Frequency count analysis of the socio-demographic characteristics of the respondents.

Variables	Experimental group (*n* = 86)		Control group (*n* = 83)	
	Frequency (*n*)	%	Frequency (*n*)	%
**Gender**				
Male	29	33.7	32	38.6
Female	57	66.3	51	61.4
**Age (years)**				
Less than 25	3	3.5	5	6.0
25–34	7	8.1	8	9.6
35–44	37	43.0	45	54.2
45 and above	39	45.3	25	30.1
**Religion**				
Christianity	51	59.3	54	65.1
Islam	29	33.7	26	31.3
Traditional	1	1.2	1	1.2
Others	5	5.8	2	2.4
**Ethnicity**				
Yoruba people	63	73.3	65	78.3
Hausa people	5	5.8	5	6.0
Igbo people	6	7.0	4	4.8
Others	12	4.0	9	10.8
**Teaching experience (years)**				
Less than 5	3	3.5	2	2.4
5–10	20	23.3	16	19.3
11–15	50	58.1	52	62.7
Above 15	13	15.1	13	15.7
**Educational status**				
NCE	3	3.5	2	2.4
Degree	63	73.3	55	66.3
Master	18	20.9	21	25.3
PhD	2	2.3	5	6.0
Total	86	100.0	83	100.0

NCE, Nigerian Certificate in Education; PhD, Doctor of Philosophy.

Ethnic distribution indicates a predominance of Yoruba respondents, making up 73.3% of the experimental group and 78.3% of the control group, while Hausa and Igbo ethnicities have relatively lower representation. Teaching experience reveals a concentration of respondents within the 11–15 years range, with 58.1% in the experimental group and 62.7% in the control group, signifying a considerable level of experience among participants. The educational status of respondents highlights that the majority hold a degree qualification, comprising 73.3% of the experimental group and 66.3% of the control group. A higher proportion of respondents in the control group (6.0%) possess a PhD compared to the experimental group (2.3%).

The descriptive analysis of the physical and physiological assessment of respondents in both the experimental and control groups reveals variations in their weight, height, body mass index (BMI), blood sugar and blood pressure (see Table 2). Regarding weight, a higher proportion of respondents in both groups fell within the 71 kg – 80 kg range (39.5% in the experimental group and 45.8% in the control group). However, more respondents in the experimental group had a weight of 80 kg and above (30.2%) compared to the control group (21.7%). In terms of height, a majority of respondents in both groups were above 1.70 m, with a slightly higher percentage in the experimental group (81.4%) than in the control group (74.7%). This indicates that most respondents were relatively tall. The BMI distribution shows that a significant proportion of respondents had a BMI above 30 kg/m^2^, indicating obesity, with the experimental group recording a higher percentage (50.0%) compared to the control group (41.0%). This suggests a higher prevalence of obesity among the experimental group.

**TABLE 2:** Descriptive analysis of the physical and physiological assessment of the respondents.

Variables	Experimental group (*n* = 86)		Control group (*n* = 83)	
	Frequency (*n*)	%	Frequency (*n*)	%
**Weight**				
Less than 50 kg	4	4.7	3	3.6
50 kg – 60 kg	12	14.0	11	13.3
61 kg – 70 kg	10	11.6	13	15.7
71 kg – 80 kg	34	39.5	38	45.8
80 kg and above	26	30.2	18	21.7
**Height**				
Less than 1.60 m	4	4.7	5	6.0
1.60 m – 1.70 m	12	14.0	16	19.3
Above 1.70 m	70	81.4	62	74.7
**Body mass index**				
Less than 20 kg/m^2^	3	3.5	3	3.6
20 kg/m^2^ – 25 kg/m^2^	8	9.3	13	15.7
26 kg/m^2^ – 30 kg/m^2^	32	37.2	33	39.8
Above 30 kg/m^2^	43	50.0	34	41.0
**Blood sugar**				
Less than 90 mg/dL	5	5.8	4	4.8
90 mg/dL – 110 mg/dL	9	10.5	16	19.3
111 mg/dL – 130 mg/dL	38	44.2	36	43.4
Above 130 mg/dL	34	39.5	27	32.5
**Blood Pressure**				
Normal (not more than 120/80 mmHg)	25	29.1	18	21.7
High normal (> 120/80 mmHg but < 140/90 mmHg)	39	45.3	41	49.4
Hypertension (> 140/90 mmHg)	22	25.6	24	28.9
Total	86	100.0	83	100.0

Furthermore, the blood sugar levels indicate that the majority of respondents in both groups had blood sugar levels within the 111 mg/dL – 130 mg/dL range (44.2% in the experimental group and 43.4% in the control group). However, a notable proportion of respondents had blood sugar levels above 130 mg/dL, with the experimental group recording a higher percentage (39.5%) compared to the control group (32.5%), which may suggest a higher risk of hyperglycaemia among the experimental group. Regarding blood pressure, a considerable number of respondents had high normal blood pressure (> 120/80 mmHg but < 140/90 mmHg), with 45.3% in the experimental group and 49.4% in the control group. However, a slightly higher proportion of respondents in the control group (28.9%) had hypertension (> 140/90 mmHg) compared to the experimental group (25.6%). This suggests a relatively higher risk of hypertension in the control group.

The categorisation of preventive practices was based on composite scores derived from summing responses to Likert scale items, where higher scores reflected more consistent engagement in positive health behaviours. For both hypertension and diabetes, the 4-point scale (‘Always’ = 4, ‘Sometimes’ = 3, ‘Rarely’ = 2, ‘Never’ = 1) allowed for quantification of frequency, with total scores categorised into good (35–40), intermediate (25–34) and poor (< 25) practice to distinguish levels of adherence. Composite scores were calculated by adding item responses, ensuring that higher totals indicated stronger preventive practice. Although this adapted scale provided a structured way of assessing practices, it was not validated specifically within this population.

The pre-intervention self-reported preventive practices of NCDs, specifically hypertension and diabetes, among teachers in both the experimental and control groups (Table 3) indicate a universally poor level of adherence to recommended preventive measures. For hypertension, all participants in both groups (100%) reported poor preventive practices, with none achieving intermediate or good practice scores. The mean scores for hypertension preventive practices were mean ± standard deviation (s.d.) = 15.06 ± 2.60 in the experimental group and mean ± s.d. = 15.02 ± 2.59 in the control group, suggesting a homogenous baseline level of poor engagement in preventive behaviours related to hypertension management. This finding underscores the necessity of an intervention aimed at improving awareness and practice of hypertension prevention strategies among teachers.

**TABLE 3:** Pre-intervention self-reported preventive practices scores for hypertension and diabetes.

Pre-intervention	Practice category	Experimental group			Control group		
		Frequency (*n*)	%	Mean ± s.d.	Frequency (*n*)	%	Mean ± s.d.
Pre-intervention self-reported preventive practices of hypertension		-	-	15.06 ± 2.60	-	-	15.02 ± 2.59
	Poor practice (10–24)	86	100	-	83	100	-
	Intermediate practice (25–34)	0	0	-	0	0	-
	Good practice (35–40)	0	0	-	0	0	-
Pre-intervention self-reported preventive practices of diabetes		-	-	12.64 ± 1.35	-	-	12.70 ± 1.27
	Poor practice (10–24)	86	100	-	83	100	-
	Intermediate practice (25–34)	0	0	-	0	0	-
	Good practice (35–40)	0	0	-	0	0	-
Pre-intervention self-reported preventive practices of NCD (hypertension and diabetes)		-	-	27.70 ± 2.83	-	-	27.72 ± 2.71
	Poor practice (20–49)	86	100	-	83	100	-
	Intermediate practice (50–69)	0	0	-	0	0	-
	Good practice (70–80)	0	0	-	0	0	-

NCD, non-communicable disease; s.d., standard deviation.

Similarly, the self-reported preventive practices for diabetes followed the same pattern (Table 3), with all participants (100%) in both groups falling within the poor practice category. No teachers in either group reported intermediate or good practices, further emphasising the lack of proactive measures taken to prevent diabetes. The mean scores for diabetes preventive practices were mean ± s.d. = 12.64 ± 1.35 and mean ± s.d. = 12.70 ± 1.27 for the experimental and control groups, respectively, indicating slight variation but still reflecting low levels of preventive engagement. These findings suggest that, prior to any intervention, there was no significant difference between the two groups in terms of diabetes prevention efforts, reinforcing the need for targeted health education and behavioural change strategies to enhance diabetes prevention among teachers.

**FIGURE 2:**
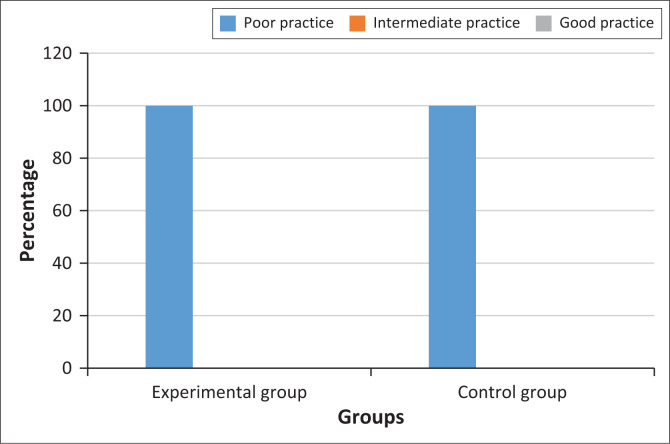
Bar chart showing pre-intervention self-reported preventive practices scores for hypertension and diabetes.

When considering the overall preventive practices for both hypertension and diabetes combined (Table 3), the pattern remains consistent, with all participants (100%) in both groups categorised as having poor preventive practices. The mean scores for combined NCD preventive practices were mean ± s.d. = 27.70 ± 2.83 in the experimental group and mean ± s.d. = 27.72 ± 2.71 in the control group, further demonstrating the absence of any meaningful engagement in preventive health behaviours (see Figure 2).

The results presented in Table 4 demonstrate a stark contrast between the experimental and control groups regarding their post-intervention self-reported preventive practices for hypertension and diabetes. In the intervention group, none of the participants reported poor practices for hypertension management post-intervention, whereas all participants (100.0%) in the control group remained in the poor practice category. A substantial proportion of the intervention group (73.3%) reported intermediate practice levels, while 26.7% achieved good practice levels. The mean practice score for hypertension in the intervention group (mean ± s.d. = 32.84 ± 2.45) was significantly higher than that of the control group (mean ± s.d. = 15.35 ± 2.17), suggesting that the intervention had a notable impact on improving preventive practices for hypertension.

**TABLE 4:** Post-intervention self-reported preventive practices scores for hypertension and diabetes.

Post-intervention	Practice category	Experimental group			Control group		
		Frequency (*n*)	%	Mean ± s.d.	Frequency (*n*)	%	Mean ± s.d.
Post-intervention self-reported preventive practices of hypertension		-	-	32.84 ± 2.45	-	-	15.35 ± 2.17
	Poor practice (10–24)	0	0.0	-	83	100.0	-
	Intermediate practice (25–34)	63	73.3	-	0	0.0	-
	Good practice (35–40)	23	26.7	-	0	0.0	-
Post-intervention self-reported preventive practices of diabetes		-	-	32.94 ± 1.74	-	-	14.14 ± 2.49
	Poor practice (10–24)	0	0.0	-	83	100.0	-
	Intermediate practice (25–34)	74	86.0	-	0	0.0	-
	Good practice (35–40)	12	14.0	-	0	0.0	-
Post-intervention self-reported preventive practices of NCD (hypertension and diabetes)		-	-	65.78 ± 2.71	-	-	29.49 ± 3.13
	Poor practice (20–49)	0	0.0	-	83	100.0	-
	Intermediate practice (50–69)	83	96.5	-	0	0.0	-
	Good practice (70–80)	3	3.5	-	0	0.0	-

NCD, non-communicable disease; s.d., standard deviation.

Similarly, for diabetes prevention, none of the participants in the intervention group remained in the poor practice category after the intervention, whereas all participants in the control group continued to report poor practices (100.0%). The majority of the experimental group (86.0%) attained intermediate practice levels, and a smaller proportion (14.0%) demonstrated good practices. The mean score for diabetes preventive practices in the experimental group (mean ± s.d. = 32.94 ± 1.74) was considerably higher than that of the control group (mean ± s.d. = 14.14 ± 2.49), further reinforcing the effectiveness of the intervention in promoting better preventive practices for diabetes management. The relatively low standard deviation in the experimental group suggests a consistent improvement in practice among participants.

**FIGURE 3:**
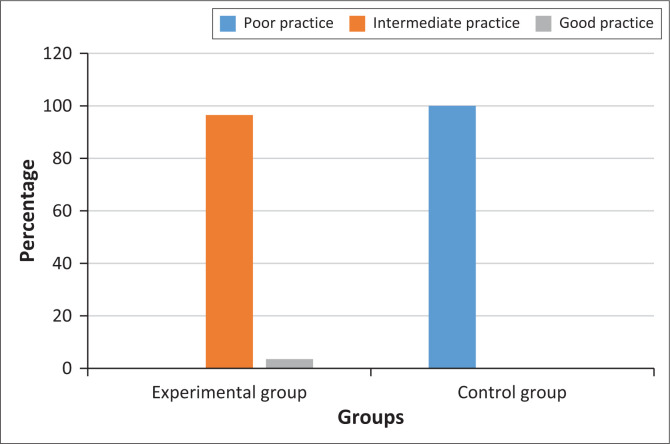
Bar Chart showing post-intervention self-reported preventive practices scores for hypertension and diabetes.

When considering the overall self-reported preventive practices for both hypertension and diabetes combined, the experimental group’s performance was significantly better than that of the control group. None of the experimental group participants reported poor practices, whereas all participants in the control group (100.0%) remained in the poor category. An overwhelming majority (96.5%) of the experimental group demonstrated intermediate practice levels, while a small fraction (3.5%) achieved good practice levels. The mean preventive practice score for the experimental group (mean ± s.d. = 65.78 ± 2.71) was more than twice that of the control group (mean ± s.d. = 29.49 ± 3.13), highlighting the significant impact of the intervention (see Figure 3).

### Hypotheses testing

**Ho1:** There is no significant difference between the mean scores of teachers in the experimental and control group before intervention on self-reported preventive practices of hypertension and diabetes.

Table 5 shows that the *t*_cal_ value of 0.059 is not significant because the *p*-value (0.953) > 0.05 at 0.05 level of significance. This implies that null hypothesis is not rejected. Hence, there was no significant difference between the mean scores of teachers in the experimental and control groups before intervention on self-reported preventive practices of hypertension and diabetes.

**TABLE 5:** *t*-test analysis for the difference in the self-reported preventive practices scores for hypertension and diabetes between teachers before intervention.

Variations	*n*	Mean	s.d.	*df*	*t*_cal_	*p*-value (Sig)	Effect size (Cohen’s *d*)
Experimental group	86	27.70	2.83	167	0.059	0.953	−0.0072
Control group	83	27.72	2.71	-	-	-	-

Note: *p* > 0.05.

Sig, significance; s.d., standard deviation; *df*, degree of freedom.

The effect size (Cohen’s *d*) of –0.0072, as presented in Table 5, indicates a negligible effect between the experimental and control groups regarding their self-reported preventive practices for hypertension and diabetes before the intervention. This suggests that the difference in the mean scores (27.70 for the experimental group and 27.72 for the control group) is virtually non-existent and does not have a meaningful impact on the preventive practices of the two groups. Therefore, the effect size reinforces that any observed difference in self-reported practices is minimal and not practically significant.

**Ho2:** There is no significant difference between the mean scores of teachers in the experimental and control group after intervention on self-reported preventive practices of hypertension and diabetes.

Table 6 shows that the *t*_cal_ value of 80.656 is significant because the *p*-value (0.000) < 0.05 at 0.05 level of significance. This implies that null hypothesis is rejected. Hence, there was significant difference between the mean scores of teachers in the experimental and control groups after intervention on self-reported preventive practices of NCD (hypertension and diabetes).

**TABLE 6:** *t*-test analysis for the difference in the self-reported preventive practices scores for hypertension and diabetes between teachers after intervention.

Variations	*n*	Mean	s.d.	*df*	*t*_cal_	*p*-value (Sig)	Effect size (Cohen’s *d*)
Experimental group	86	65.78	2.71	167	80.656*	0.000	12.41 (11.05–13.77)
Control group	83	29.49	3.13	-	-	-	-

Sig, significance; s.d., standard deviation; *df*, degree of freedom.

* , *p* < 0.05.

The effect size of Cohen’s *d* is 12.41, with a 95% confidence interval ranging from 11.05 to 13.77. This large effect size indicates a substantial difference in the self-reported preventive practices of NCD (hypertension and diabetes) between the experimental and control groups after the intervention. The experimental group, with a mean score of 65.78, demonstrated significantly improved practices compared to the control group, which had a mean score of 29.49. The large effect size suggests that the intervention had a highly positive impact on the teachers’ preventive practices for hypertension and diabetes.

## Discussion

The findings of this study indicate a universally poor level of adherence to preventive practices for NCDs, specifically hypertension and diabetes, among teachers in both the experimental and control groups before intervention. This is evidenced by the fact that all participants (100%) reported poor preventive practices, with none achieving intermediate or good practice scores. These findings highlight a crucial gap in the adoption of preventive strategies, reinforcing the necessity for intervention programmes aimed at improving health awareness and encouraging better lifestyle choices among teachers.

The findings align with existing literature that underscores the significant role of behavioural risk factors in the prevalence of hypertension and diabetes. Yu et al.^10^ identified tobacco use, unhealthy diets, physical inactivity and excessive alcohol consumption as key contributors to the high risk of these diseases. The universally poor self-reported preventive practices observed in this study reflect a lack of awareness and engagement in mitigating these risk factors. Furthermore, some studies emphasised that lifestyle modification has the potential to reduce the risk of diabetes by up to 70%, which suggests that the absence of proper preventive measures among teachers places them at significant risk of developing these conditions.^3,10,23^ Similarly, Kilama et al.^11^ highlighted the importance of early detection through routine screening, counselling and medication adherence, strategies that appear to be absent among the study participants.

Misconceptions and poor knowledge about hypertension and diabetes among Nigerians have been documented by some studies, which further support the study’s findings.^11,16,24^ The reported poor preventive practices among teachers could be linked to a general lack of awareness regarding NCD prevention. Anyanti et al.^20^ found that a significant portion of Nigerians engage in unhealthy lifestyle behaviours, with 25% not engaging in physical activity, 13% consuming harmful levels of alcohol and 6% being cigarette smokers. The prevalence of these unhealthy behaviours is consistent with the poor engagement in preventive measures observed in the study, suggesting that broader societal factors may contribute to the lack of proactive health management among teachers.

After intervention, the findings of the study revealed a significant improvement in post-intervention self-reported preventive practices for hypertension and diabetes among participants in the experimental group compared to those in the control group. These results suggest that the intervention was highly effective in fostering behavioural changes necessary for preventing and managing these chronic conditions. The significant improvement in the experimental group’s preventive practices for hypertension and diabetes is consistent with the study that lifestyle-related risk factors such as physical inactivity, poor diet, tobacco use and harmful alcohol consumption contribute substantially to hypertension and diabetes.^10^ Yu et al.^10^ further highlight that lifestyle modifications can reduce diabetes risk by as much as 70%, reinforcing the effectiveness of the intervention in this study. The increase in good and intermediate practice levels among intervention participants suggests that they may have adopted healthier lifestyles, which is critical in preventing these conditions. The findings also align with another study that emphasises the importance of early detection, routine screening, counselling and medication adherence in managing hypertension and diabetes.^11^ Since the experimental group exhibited better preventive practices post-intervention, it can be inferred that the intervention may have incorporated educational components addressing these aspects.

However, the control group’s persistent poor practices (100%) contrast with findings from some studies, which reported that 52.35% of participants engaged in physical activity, albeit with limited knowledge of risk factors.^21^ This suggests that without targeted interventions, individuals may continue engaging in unhealthy behaviours because of poor awareness. Another study further corroborates this, as it found that only 18% of respondents were aware of risk factors associated with hypertension and diabetes, indicating a general knowledge deficit.^19^

Moreover, the improvements in self-reported preventive practices reflect behavioural modifications influenced by increased knowledge and perceived health risks.^14^ According to the Health Belief Model,^25^ individuals are more likely to adopt healthy behaviours when they perceive a condition as serious, understand its risk factors and recognise the benefits of preventive actions.^24^ The intervention helped teachers connect knowledge with action, leading to sustained behavioural improvements. Additionally, the approach in the intervention facilitated sustained engagement and reinforcement of healthy behaviours. Teachers who actively participated in group discussions and practical sessions were more likely to implement what they learned, further demonstrating the importance of interactive and experiential learning in health education.^21^

This study contributes significantly to the field of health education by providing empirical evidence that structured health interventions effectively enhance teachers’ knowledge and preventive practices regarding hypertension and diabetes. It highlights critical gaps in teachers’ awareness and preventive behaviours before the intervention, reinforcing the need for continuous health literacy initiatives within the education sector. The study justifies integrating NCD education into teacher training programmes and professional development initiatives, demonstrating that improved knowledge leads to better preventive practices. Additionally, it reveals that socio-demographic characteristics do not significantly influence teachers’ knowledge and practices, suggesting that factors such as institutional health policies, access to healthcare and digital health literacy should be explored further. These findings contribute to the growing body of literature on workplace health interventions and their impact on disease prevention, providing valuable insights for educators, healthcare professionals and policymakers.

A key limitation of this study lies in its reliance on self-reported data, which may be subject to social desirability bias or recall inaccuracies, potentially influencing the validity of the findings. Additionally, the short-term follow-up limits the ability to assess the sustained impact of the intervention over time. Furthermore, the study did not control for potential confounding variables such as participants’ baseline health literacy, socio-economic status or prior exposure to health education, which may have influenced their preventive practices independently of the intervention.

Another limitation of the study is the constrained generalisability of the findings because of the sampling approach. The sample was drawn from only two LGAs in Lagos State, and the selection of schools was purposive rather than random. As a result, the findings may not accurately represent the broader population of teachers across other LGAs in Lagos or different regions of Nigeria. Variations in demographic, institutional and cultural factors could influence preventive health practices, and thus caution is needed in applying these results beyond the study context. Future studies should consider larger, more diverse and randomly selected samples to enhance generalisability.

The limitations of this study affect interpretation by introducing potential biases and restricting the confidence with which findings can be generalised. The reliance on self-reported data may have inflated or understated responses because of recall lapses or social desirability, reducing the accuracy of conclusions. Similarly, the short-term follow-up prevents an understanding of whether the observed changes in preventive practices can be maintained over time, thereby limiting insights into the intervention’s long-term effectiveness. The purposive sampling of schools within only two LGAs in Lagos State narrows the representativeness of the results, meaning they may not reflect the diversity of teachers’ experiences and practices across different regions of Nigeria. To address these issues, future research should incorporate more representative and randomly selected samples across multiple LGAs or states while also extending the duration of follow-up to track sustained behavioural change. Additionally, controlling for key confounders would help isolate the true impact of the intervention and strengthen the robustness of conclusions.

The study’s implications extend to nursing practice, teaching, research and health policy. It underscores the role of nurses in promoting preventive healthcare by incorporating routine health education and screening into their practice, particularly in community and occupational health settings. For teaching, it advocates for integrating NCD education into teacher training curricula and professional development programmes, ensuring teachers can foster a culture of health consciousness in schools. The research findings pave the way for future studies on the long-term effects of NCD awareness programmes and the effectiveness of different instructional methods. In terms of policy, the study calls for the integration of health education into teacher training, routine school health screenings and stronger collaboration between health and education sectors.

Based on the findings, the study recommended that:

- Schools should partner with health agencies to provide routine health screenings and awareness programmes for teachers. Regular monitoring of blood pressure and blood glucose levels will facilitate early detection of hypertension and diabetes, encouraging timely intervention and lifestyle modifications.
- Schools should implement workplace wellness programmes that promote healthy lifestyles, including exercise sessions, dietary guidance and stress management techniques. Creating a supportive environment that encourages physical activity and healthy eating will enhance preventive practices among teachers.
- Future health promotion programmes should formally integrate teachers as key health educators within schools, leveraging their position to disseminate preventive messages to students.

## Conclusion

The study found the critical gap in preventive practices regarding hypertension and diabetes among teachers prior to the intervention. Both the experimental and control groups demonstrated poor engagement in preventive behaviours, underscoring the urgent need for targeted health education. However, the post-intervention results reveal a remarkable improvement in the experimental group’s self-reported preventive practices, signifying the effectiveness of the intervention in enhancing teachers’ proactive management of these diseases.
